# BDNF-TrkB/proBDNF-p75^NTR^ pathway regulation by lipid emulsion rescues bupivacaine-induced central neurotoxicity in rats

**DOI:** 10.1038/s41598-023-45572-8

**Published:** 2023-10-26

**Authors:** Danting Jia, Fang Wang, Zhixia Bai, Xuexin Chen

**Affiliations:** 1 Department of Anesthesiology, General Hospital of Ningxia Medical University, Yinchuan, 750004 Ningxia China; 2 Department of Anaesthesiology, People’s Hospital of Ningxia Hui Autonomous Region, Yinchuan, 750002 Ningxia China

**Keywords:** Cell biology, Neuroscience, Medical research

## Abstract

Bupivacaine (BPV) can cause severe central nervous system toxicity when absorbed into the blood circulation system. Rapid intravenous administration of lipid emulsion (LE) could be used to treat local anaesthetic toxicity. This study aimed to investigate the mechanism by which the BDNF-TrkB/proBDNF-p75^NTR^ pathway regulation by LE rescues BPV induced neurotoxicity in hippocampal neurons in rats. Seven- to nine-day-old primary cultured hippocampal neurons were randomly divided into 6 groups: the blank control group (Ctrl), the bupivacaine group (BPV), the lipid emulsion group (LE), the bupivacaine + lipid emulsion group (BPV + LE), the bupivacaine + lipid emulsion + tyrosine kinase receptor B (TrkB) inhibitor group (BPV + LE + K252a), the bupivacaine + lipid emulsion + p75 neurotrophic factor receptor (p75^NTR^) inhibitor group (BPV + LE + TAT-Pep5). All hippocampal neurons were incubated for 24 h, and their growth state was observed by light microscopy. The relative TrkB and p75^NTR^ mRNA levels were detected by real-time PCR. The protein expression levels of brain-derived neurotrophic factor (BDNF), proBDNF, TrkB, p75^NTR^ and cleaved caspase-3 were detected by western blotting. The results showed that primary hippocampal neuron activity was reduced by BPV. As administration of LE elevated hippocampal neuronal activity, morphology was also somewhat improved. The protein expression and mRNA levels of TrkB and p75^NTR^ were decreased when BPV induced hippocampal neuronal toxicity, while the expression of BDNF was increased. At the same time, BPV increased the original generation of cleaved caspase-3 protein content by hippocampal neurons, while the content of cleaved caspase-3 protein in hippocampal neurons cotreated with LE and BPV was decreased. Thus, this study has revealed LE may reduce apoptosis and promote survival of hippocampal neurons by regulating the BDNF-TrkB pathway and the proBDNF-p75^NTR^ pathway to rescue BPV induced central neurotoxicity in rats.

## Introduction

The advantages of local anaesthesia techniques throughout the perioperative period have been recognized^1,2^. Although many measures have been taken to improve the safety of local anaesthesia^3,4^, body absorption and the systemic toxicity of local anaesthetics remain concerns. Convulsion and cardiac arrest are the most harmful acute complications of the systemic toxicity of local anaesthetics. Central nervous system (CNS) toxicity, manifesting as convulsions, appears earlier than cardiocirculatory system toxicity, and the toxic dose for the CNS is lower than the dose needed to cause cardiac arrest^5,6^. Therefore, the prevention and treatment of CNS toxicity are particularly important when using local anaesthetics.

Clinically, when local anaesthetics caused CNS toxicity in the past, sedatives were mainly used to treat or relieve the toxic symptoms, but the incidence of respiratory inhibition was relatively high. Once anoxia occurs, it will aggravate CNS toxicity and increase its sequelae. In addition, sedatives themselves have adverse effects on neurons and potentially neurotoxic effects on the brain^7^. In recent years, there has been increasing evidence supporting the use of lipid emulsion (LE) in the treatment of local anaesthesia poisoning, especially bupivacaine poisoning. a commonly used local anaesthetic in clinical practice^5,6,8–11^. Cell experiments and isolated heart model experiments have shown that LE treatment can reduce BPV-induced cell death and promote the recovery of cardiac function^12,13^. This may be related to the fact that LE significantly improves the mitochondrial function of cardiomyocytes and reverses BPV-induced apoptosis^14^. However, existing studies have given more attention to the cardiotoxicity of BPV^15–17^, and less attention has been given to its CNS toxicity mechanism. The occurrence and development of CNS toxic convulsions are closely related to the hippocampus^18–20^. Convulsions caused by local anaesthesia can increase the apoptosis of hippocampal neurons in rats^20^. Although laboratory data and case reports support treating bupivacaine CNS toxicity with LE, and the positive effect is clear^6,8^, the exact mechanism has not been uncovered.

It is generally believed that the CNS toxicity of bupivacaine occurs because bupivacaine blocks the inhibitory pathway of the brain, resulting in the relative activation of the excitatory pathway. If the concentration of bupivacaine in the blood continues to rise, the inhibitory and excitatory pathways will be inhibited at the same time, thus inhibiting the entire CNS^21^. During this process, there is an imbalance between excitatory amino acids and inhibitory amino acids in the brain, causing disorders of brain nerve function. In the brain, the balance between excitatory amino acids and inhibitory amino acids is mainly due to the binding of BDNF to TrkB and/or proBDNF to p75^NTR^, which activate the regulation of downstream PI3K/Akt or Ras/MAPK signalling pathways. However, it has been reported that bupivacaine can inhibit the PI3K/Akt signalling pathway and lead to the occurrence of neuronal apoptosis^22,23^. Therefore, we speculate that bupivacaine can activate the downstream pathway through its interaction with BDNF, TrkB, and/or proBDNF-p75^NTR^, causing the excitatory amino acids and inhibitory amino acids in the brain to be out of balance, resulting in CNS toxicity, which eventually leads to cell apoptosis or death.

Based on previous studies and literature reports, it has been confirmed that the CNS toxicity of BPV is caused by an imbalance in the expression of excitatory amino acids, inhibitory amino acids, and their corresponding receptors in the brain. LE can improve the imbalance and play a role in treating CNS toxicity caused by bupivacaine^24^. Therefore, we sought to answer the following question: does LE reverse the CNS toxicity induced by BPV by regulating BDNF-TrkB/proBDNF-p75^NTR^?

This experiment utilized primary hippocampal neurons as the research object to study the relationship between the BDNF-TrkB/proBDNF-p75^NTR^ pathway and BPV-induced hippocampal neuron toxicity, and to determine whether it participated in the process of LE treatment of BPV-induced CNS toxicity, to provide a more solid theoretical basis for its wide clinical applications.

## Materials and methods

### Chemicals and antibodies

Bupivacaine hydrochloride injection was purchased from Zhaohui Pharmaceutical (Shanghai, China). 20% lipid emulsion was purchased from the Kelun industry group (Sichuan, China). TAT-Pep5 was obtained from Merck Millipore (Germany). Poly-L-lysine solution and K252a were purchased from Sigma (USA). Trypsin–EDTA (0.25%), Dulbecco's modified Eagle's medium (DMEM), neurobasal medium, B27 supplement, and foetal bovine serum (FBS) were purchased from Gibco (USA). BDNF and TrkB antibodies were purchased from Abcam (USA). The proBDNF antibody was purchased from Novusbio (USA). Cleaved Caspase-3 (D175) was purchased from Signalway Antibody (SAB, USA). p75^NTR^ was purchased from CST(USA). Biotinylated goat anti-mouse IgG whole antibody and β-actin were obtained from Santa Cruz (USA).

### Primary hippocampal neuron culture

SD rats were sterilized with 75% ethanol within 24 h of birth. The pups were decapitated with fresh sterile scissors and the removed head was placed on sterile gauze. The hippocampal tissue from both sides was separated quickly. The hippocampal tissues were placed into 0.25% pancreatic enzyme and digested for 20 min at 37 ℃. After digestion, the tissue was washed and fixed. After collecting the supernatant, the cells were extracted from the supernatant. Next, cells were plated in 96-well plates and 6- well plates that were treated with polylysine, cleaned with inoculation fluid 4 h after inoculation, and replaced with neuronal complete medium. The next day, half the amount of liquid was changed, and the culture was replaced with cytarabine (complete medium and a final concentration of 2.5 μg/ml cytarabine) after 48 h. Then, the culture fluid was changed every 2–3 d, half the amount of liquid was changed each time, and the liquid was cultured for 7–9 days and reserved.

### Experimental classification

Hippocampal neurons cultured for 7–9 days were divided into six groups: control, the bupivacaine group(BPV), the lipid emulsion group(LE), the bupivacaine + lipid emulsion group(BPV + LE), the bupivacaine + lipid emulsion + TrkB inhibitor group(BPV + LE + 200 nM K252a), and the bupivacaine + lipid emulsion + p75^NTR^ inhibitor group(BPV + LE + 2 μM TAT-Pep5).

### MTT assay for the activity of primary hippocampal neurons

Primary hippocampal neurons were divided into different groups and treated in 96-well plates. Next, 25 μM MTT was added to each well to a final concentration of 1 mg/ml. The wells were mixed and then cultured at 37 °C for 4 h. After that, 100 μM solution (25% SDS + 50% DMF) was added to each well and incubated overnight at 37 °C. The absorbance values of each well were read at a wavelength of 570 nm by a microplate reader, and the experiment was repeated three times.

Determining the half maximal inhibitory concentration (IC50) of BPV: Primary hippocampal neurons were treated with different concentrations of BPV for 24 h, cell viability was measured by the MTT method, and the IC50 of BPV was calculated. The concentration of the BPV stock solution was 0.75%, and the highest dosing concentration was 0.25%. It was diluted three times, and a total of 9 concentrations were set.

### Real-time PCR assay

Total RNA from cultured hippocampal neurons in each group was extracted with TRIzol reagent. First-strand cDNAs were generated using the PrimeScriptTM RT reagent kit (TaKaRa, Japan). Finally, according to the manufacturer’s recommendations, quantitative real-time PCR was performed using the SYBR premix reaction system (TaKaRa, Japan). GAPDH was used as the endogenous control. The assay was performed using fluorescence quantitative PCR instrument (Applied Biosystems, USA) to analyse the relative expression levels of mRNAs. The relative gene expression was calculated using the 2^−ΔΔCT^ method. ( The primers are listed in Table 1.)

**Table 1:** Real-time PCR primer sequence.

Primer	Sequence
Rat p75^NTR^-F	TGCTGCTGATTCTAGGGATGTC
Rat p75^NTR^-R	TTCACACACGGTCTGGTTGG
Rat TrkB-F	ACCTGCGGCACATCAATTTC
Rat TrkB-R	GGTTCGCCAGAGGGGTATTC
Rat GAPDH-F	GGCAAGTTCAACGGCACAGT
Rat GAPDH-R	TGGTGAAGACGCCAGTAGACTC

### Western blot analysis

The total protein of hippocampal neurons in each group was extracted, and the protein concentration was measured with the bicinchoninic acid (BCA) protein assay kit. Protein samples were separated by sodium dodecyl sulfate polyacrylamide gel electrophoresis (SDS‒PAGE), transferred to polyvinylidene fluoride (PVDF) membranes, blocked, and incubated with anti-BDNF (1:800), anti-proBDNF (1:200), anti- cleaved caspase-3 (1:500), anti-p75^NTR^ (1:500), or anti-TrkB (1:1000) antibodies diluted with TBST containing 1% bovine serum albumin(BSA) overnight at 4 °C. On the next day, the membrane was washed three times with TBST for 10 min each time. The membrane was incubated with an HRP-labelled secondary antibody at a dilution of 1:5000 for approximately 1 h at room temperature. After rinsing, the membranes were exposed to an X-ray film. Quantification of bands was performed by scanning the films.

### Statistical analyses

All data were analysed using SPSS 21.0 software (IBM, USA). All values are expressed as the means ± SDs. Data were analysed by one-way analysis of variance (ANOVA) to determine statistically significant differences. *P* value < 0.05 was considered significant.

### Ethics approval and consent to participate

The study was approved by the Committee on the Ethics of Animal Experiments at Ningxia Medical University (Approval No. 2015–058). Animals were cared for in accordance with the University’s standards for care and use of laboratory animals, and all procedures were performed in accordance with the revised Animals (Scientific Procedures) Act 1986. Meanwhile, ARRIVE guidelines were followed during this study.

## Results

### Effect of bupivacaine on the activity of primary hippocampal neurons

**Figure 1:**
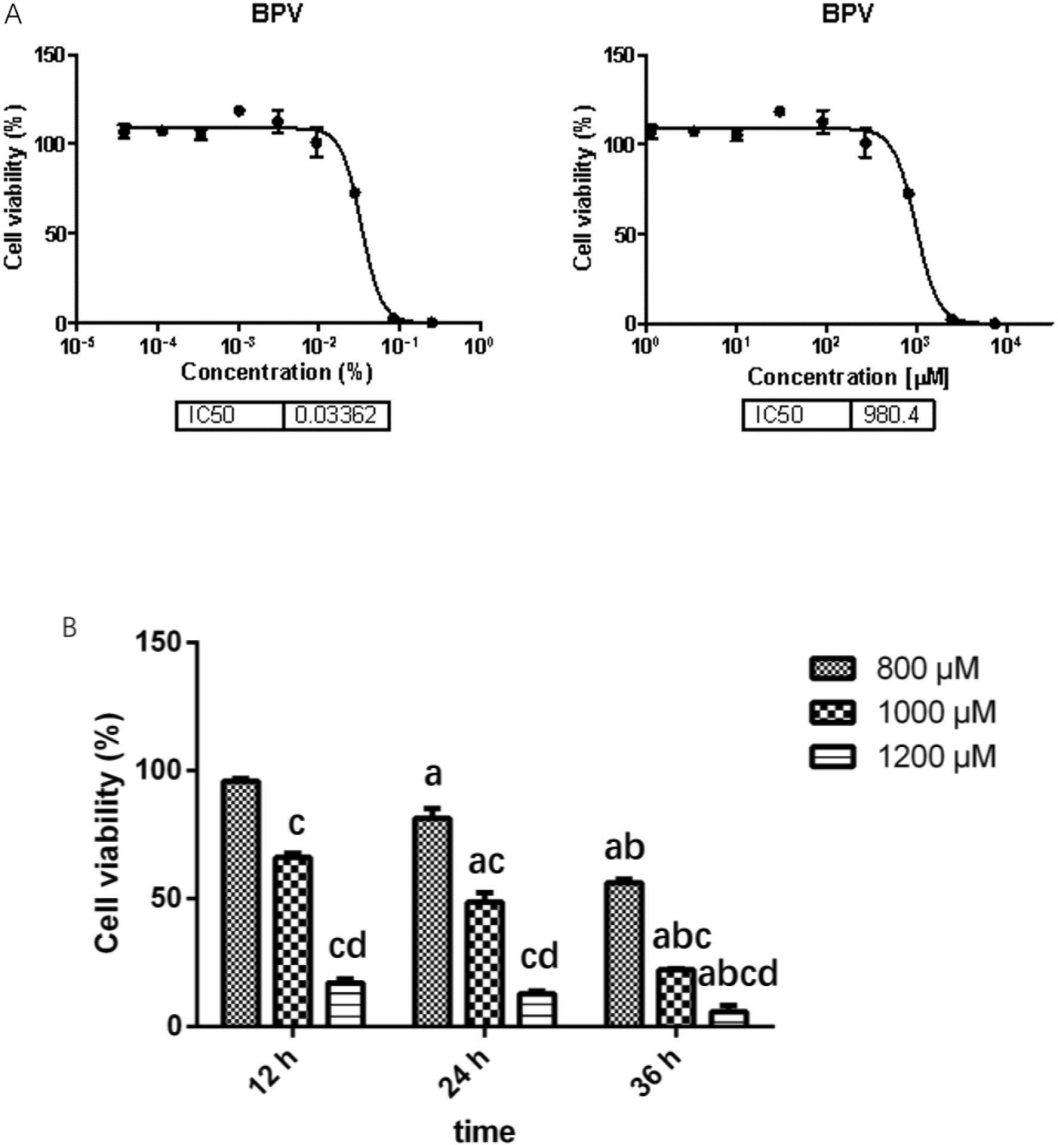
Effects of different bupivacaine concentrations and treatment times on hippocampal neurons. (**A**) The IC50 of BPV was 0.03362%, which was converted into a molar concentration of 980.4 μM. (**B**) The activity of hippocampal neurons decreased with the increase of BPV concentration and treatment time. The activity of hippocampal neurons decreased by about 50% after 24 h with 1000 μM. (*x̅* ± s, triplicate). Same BPV concentration, compared with the 12 h: ^a^*P* < 0.05; Same BPV concentration, compared with 24 h: ^b^*P* < 0.05; Same treatment time, compared with 800uM: ^c^*P* < 0.05; Same treatment time, compared with 1000uM: ^d^*P* < 0.05.

The IC50 of BPV was 0.03362%, which was converted into a molar concentration of 980.4 μM (Fig. 1A). Therefore, BPV at concentrations of 800 μM, 1000 μM, and 1200 μM was selected to treat primary hippocampal neurons for 12 h, 24 h, and 36 h, respectively. The activity of hippocampal neurons was measured by MTT assay. As shown in Fig. 1B, the activity of hippocampal neurons decreased with increasing BPV concentration; with the same concentration of BPV, the activity of hippocampal neurons decreased with increasing treatment time. The activity of hippocampal neurons decreased by approximately 50% after 24 h with 1000 μM or 1 mM BPV. Therefore, this concentration and time were selected for subsequent experiments (Fig. 1).

### Effect of LE on hippocampal neuron activity

**Figure 2:**
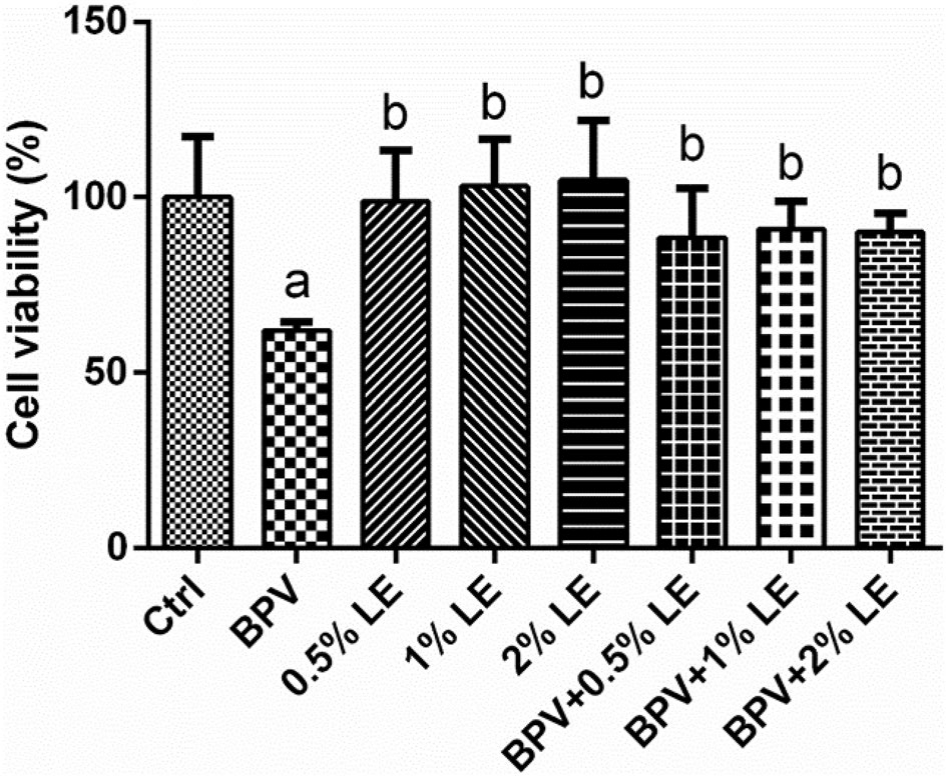
The activity of hippocampal neurons was studied using an MTT assay (1000 μM, 24 h). The BPV group’s hippocampal neuronal activity was reduced compared with the control group, (*P* < 0.05). Compared with the BPV group, the activity of hippocampal neurons in the 0.5% LE group, 1% LE group, 2% LE group, BPV + 0.5% LE group, BPV + 1% LE group, and BPV + 2% LE group was increased (P < 0.05). (*x̅* ± s, triplicate). Compare with Ctrl: ^a^*P* < 0.05; compared with group BPV: ^b^*P* < 0.05.

LE at three concentrations, 0.5%, 1%, and 2%, was selected to treat hippocampal neurons exposed to BPV and the activity of hippocampal neurons was observed. As shown in Fig. 2, compared with the control group, the BPV group’s hippocampal neuronal activity was reduced (*P* < 0.05). Compared with the BPV group, the activity of hippocampal neurons in the 0.5% LE group, 1% LE group, 2% LE group, BPV + 0.5% LE group, BPV + 1% LE group, and BPV + 2% LE group was increased (*P* < 0.05) (Fig. 2).

### Growth status of hippocampal neurons

**Figure 3:**
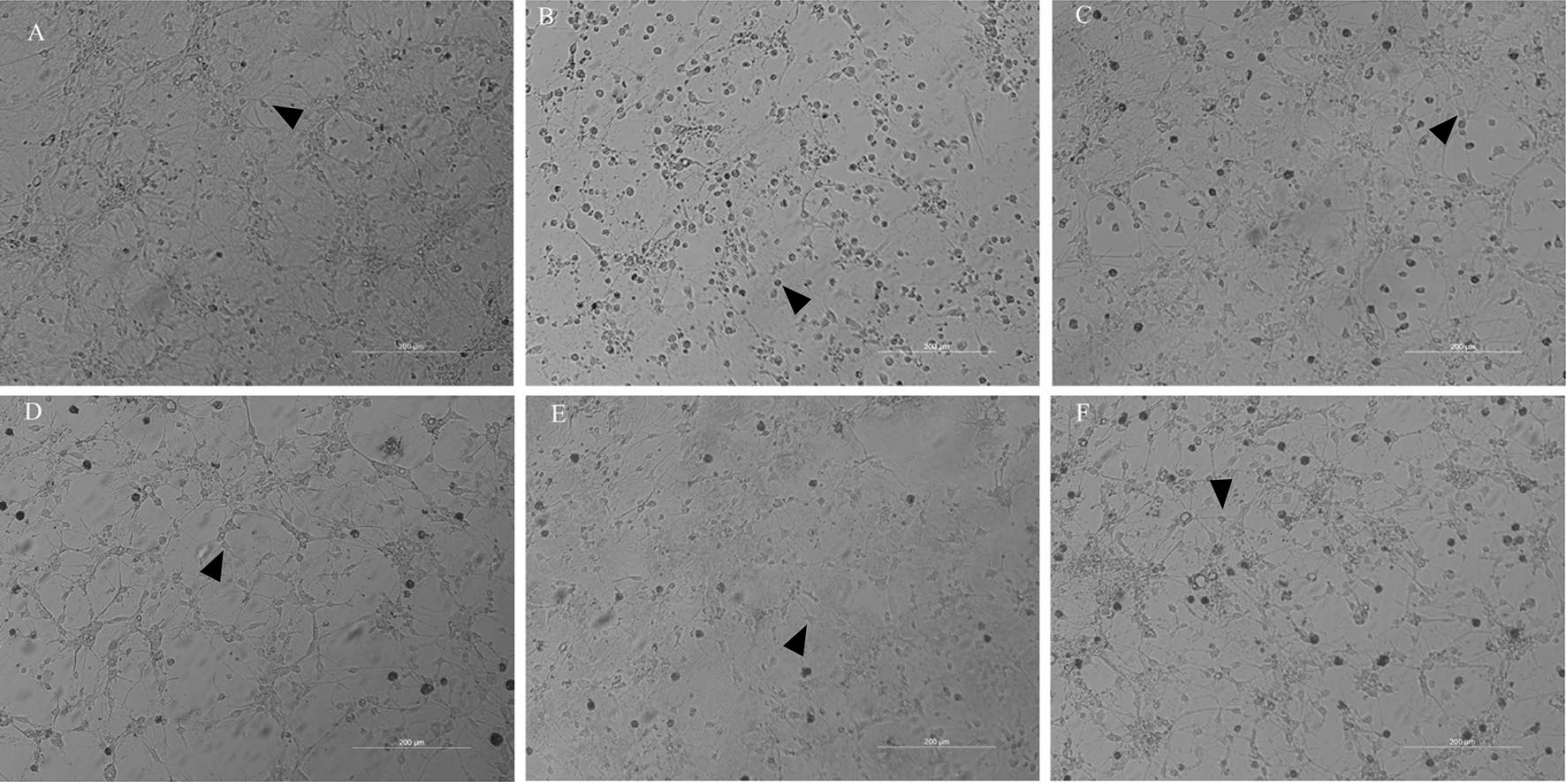
Growth status of hippocampal neurons in six groups (200×) (1000 μM, 24 h, triplicate) Ctrl group (**A**) and LE group (**C**): The hippocampal neurons were plump, with strong stereoscopic sense, thick and long protrusions, and were interwoven into a dense network. BPV group (**B**): The hippocampal neurons were wrinkled, with thin, short, or no protrusions, significantly reduced connections between neurons and no dense network formation. BPV + LE group (**D**): The hippocampal neurons were round, with a small amount of wrinkling and long protrusions, which could interact with the surrounding neurites to form a network. BPV + LE + K252a group (**E**) and BPV + LE + TAT-Pep5 (**F**) group: The hippocampal neurons were arranged into clusters, the cell body was wrinkled, and protuberances were present, which could be interwoven into a network with the surrounding neuronal protuberances. (**A**) Control; (**B**) BPV; (**C**) LE; (**D**) BPV + LE; (**E**) BPV + LE + K252a; (**F**) BPV + LE + TAT-Pep5.

As shown in Fig. 3, the hippocampal neurons of the Ctrl group and the LE group were plump, with strong stereoscopic sense and thick and long protrusions, and were interwoven into a dense network. In the BPV group, hippocampal neurons were wrinkled, with thin, short, or no protrusions, significantly reduced connections between neurons and no dense network formation. The hippocampal neurons in the BPV + LE group were round, with a small amount of wrinkling and long protrusions, which could interact with the surrounding neurites to form a network. The hippocampal neurons in the BPV + LE + K252a group and the BPV + LE + TAT-Pep5 group were arranged into clusters, the cell body was wrinkled, and protuberances were present, which could be interwoven into a network with the surrounding neuronal protuberances (Fig. 3).

### Expression of TrkB and p75^NTR^ mRNA in six groups of hippocampal neurons

**Figure 4:**
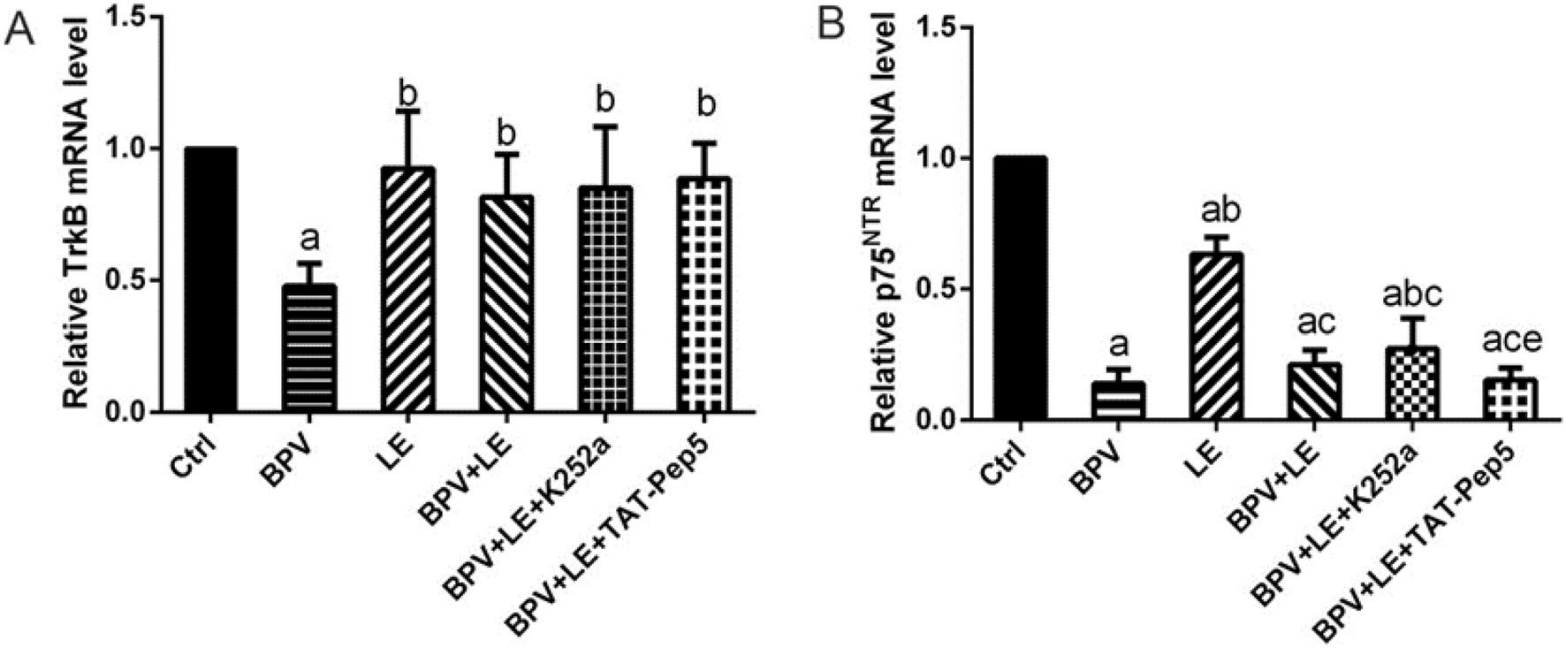
Expression of TrkB and p75^NTR^ mRNA in six groups of hippocampal neurons. (1000 μM, 24 h). The mRNA level of TrkB were decreased when BPV induced hippocampal neuronal toxicity, while the expression of BPV + LE group was increased. The mRNA level of p75^NTR^ were decreased when BPV induced hippocampal neuronal toxicity, while the expression of BPV + LE group was decreased compared with the LE group. (**A**) Comparison of TrkB mRNA expression (*x̅* ± s, triplicate); (**B**) Comparison of expression of p75^NTR^ mRNA (*x̅*  ± s, triplicate). Compared with the control: ^a^*P* < 0.05; compared with group BPV: ^b^*P* < 0.05; compared with group LE: ^c^*P* < 0.05; compared with group BPV + LE + K252a: ^e^*P* < 0.05.

TrkB mRNA expression in the six groups of hippocampal neurons is shown in Fig. 4A. Compared with that in the Ctrl group, TrkB mRNA expression in the BPV group was decreased (*P* < 0.05). Compared with the BPV group, TrkB mRNA expression was increased in the BPV + LE group, BPV + LE + K252a group, and BPV + LE + TAT-Pep5 group (*P* < 0.05). Compared with that in the LE group, TrkB mRNA expression in the BPV + LE group, BPV + LE + K252a group, and BPV + LE + TAT-Pep5 group was decreased, but the difference was not statistically significant (*P* > 0.05).

The expression of p75^NTR^ mRNA in the six groups of hippocampal neurons is shown in Fig. 4B. Compared with the control group, p75^NTR^ mRNA expression in the BPV group, LE group, BPV + LE group, BPV + LE + K252a group, and BPV + LE + TAT-Pep5 group was decreased (*P* < 0.05). Compared with the BPV group, the expression of p75^NTR^ mRNA in the LE group and the BPV + LE + K252a group was increased (*P* < 0.05), the expression of p75^NTR^ mRNA in the BPV + LE group was also increased, but the difference was not statistically significant (*P > 0.05). The expression of p75*^*NTR*^* mRNA in the BPV + LE group, B*PV + LE + K252a group, and BPV + LE + TAT-Pep5 group was decreased compared with the LE group (*P* < 0.05). The expression of p75^NTR^ mRNA increased in the BPV + LE + K252a group and decreased in the BPV + LE + TAT-Pep5 group compared with the BPV + LE group, but the difference was not statistically significant (*P* > 0.05). Compared with the BPV + LE + K252a group, the expression of p75^NTR^ mRNA in the BPV + LE + TAT-Pep5 group was decreased (*P* < 0.05) (Fig. 4).

### BDNF, proBDNF, TrkB, p75^NTR^, and Cleaved caspase-3 protein expression

**Figure 5:**
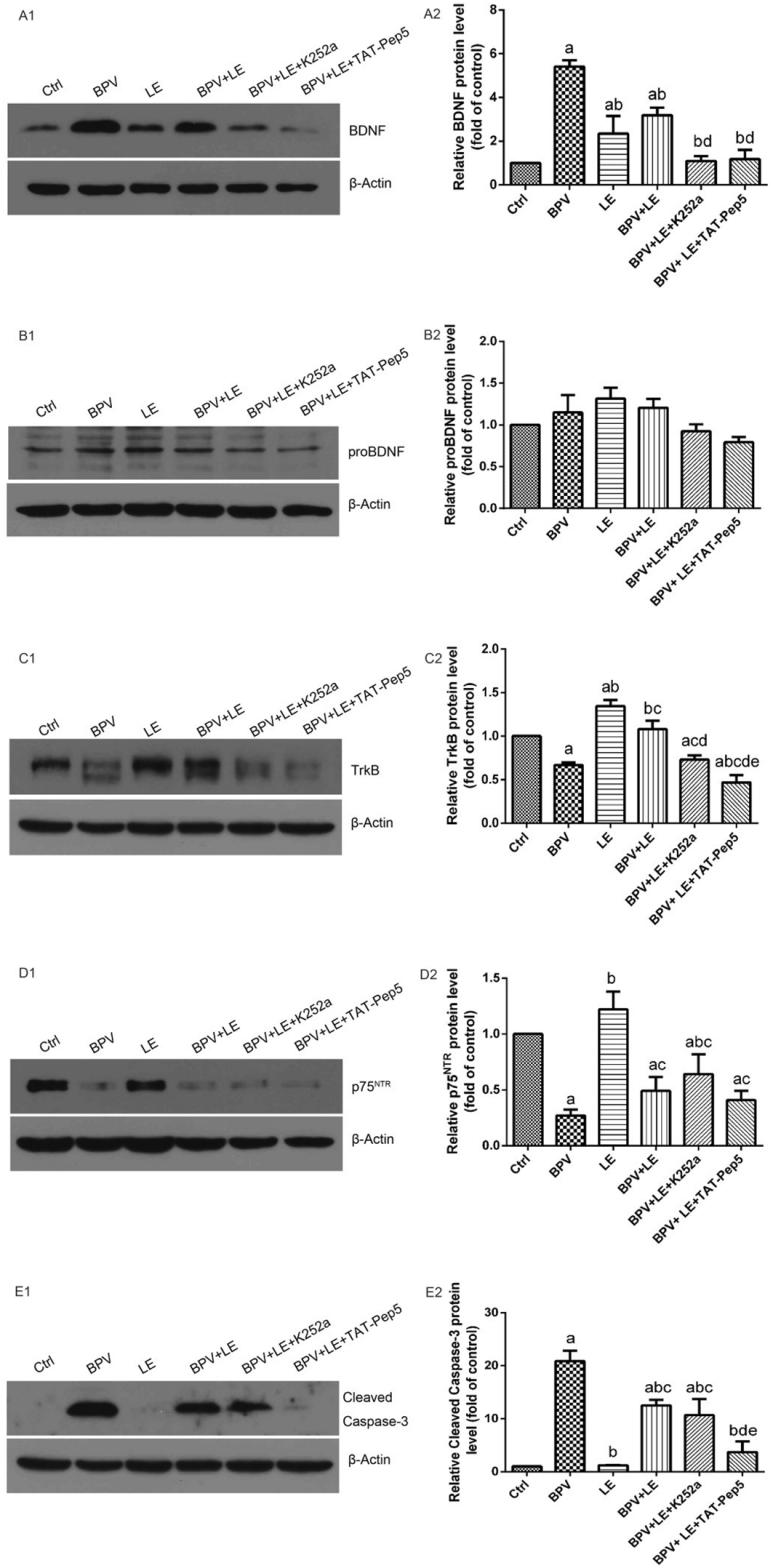
Expression of BDNF, proBDNF, TrkB, p75^NTR^, and Cleaved caspase-3 proteins in six groups of hippocampal neurons(1000 μM, 24 h) (Electrophoretic gels and blots were cropped, the samples derive from the same experiment and that gels/blots were processed in parallel.). The protein expression of TrkB and p75^NTR^ were decreased when bupivacaine induced hippocampal neuronal toxicity, while the expression of BDNF was increased and proBDNF showed no significant change. BPV increased the original generation of cleaved caspase-3 protein content by hippocampal neurons, and LE and BPV decreased the cleaved caspase-3 protein content of hippocampal neurons. A1-E1: Protein expression blot of BDNF, proBDNF, TrkB, p75^NTR^, and Cleaved caspase-3; A2-E2, The relative expression levels of BDNF, proBDNF, TrkB, p75^NTR^, and Cleaved caspase-3 proteins were standardized with internal reference to β-actin (*x̅* ± s , triplicate). Compared with the control: ^a^*P* < 0.05; compared with group BPV: ^b^*P* < 0.05; compared with group LE: ^c^*P* < 0.05; compared with group BPV + LE: ^d^*P* < 0.05; compared with group BPV + LE + K252a: ^e^*P* < 0.05.

The expression of BDNF protein in the six groups of hippocampal neurons is shown in Fig. 5, A1–A2, and the difference between the groups was statistically significant (*P* < 0.05). Compared with the Ctrl group, BDNF protein expression in the BPV group, LE group, and BPV + LE group was increased, with a statistically significant difference (*P* < 0.05). Compared with that in the BPV group, the expression of BDNF protein in the LE group, BPV + LE group, BPV + LE + K252a group, and BPV + LE + TAT-Pep5 group was decreased (*P* < 0.05). Compared with the LE group, BDNF protein expression was increased in the BPV + LE group and decreased in the BPV + LE + K252a group and BPV + LE + TAT-Pep5 group, and the difference was not statistically significant (*P* > 0.05). Compared with that in the BPV + LE group, BDNF protein expression in the BPV + LE + K252a group and the BPV + LE + TAT-Pep5 group was decreased (*P* < 0.05).

There was no significant change in the expression of proBDNF protein in the six groups of hippocampal neurons (*P* > 0.05), as shown in Fig. 5, B1 and B2.

TrkB protein expression in six groups of hippocampal neurons is shown in Fig. 5, C1–C2. Compared with the Ctrl group, TrkB protein expression in the BPV group, BPV + LE + K252a group, and BPV + LE + TAT-Pep5 group was decreased (*P* < 0.05). Compared with the BPV group, TrkB protein expression in the BPV + LE group was increased (*P* < 0.05), TrkB protein expression in the BPV + LE + K252a group was not significantly changed (*P* > 0.05), and TrkB protein expression in the BPV + LE + TAT-Pep5 group was decreased (*P* < 0.05). Compared with that in the BPV + LE group, TrkB protein expression in the BPV + LE + K252a group and BPV + LE + TAT-Pep5 group was decreased (*P* < 0.05). Compared with the BPV + LE + K252a group, TrkB protein expression in the BPV + LE + TAT-Pep5 group decreased (*P* < 0.05).

The expression of p75^NTR^ protein in the six groups of hippocampal neurons is shown in Fig. 5, D1–D2. Compared with the control group, p75^NTR^ protein expression was decreased in the BPV group, BPV + LE group, BPV + LE + K252a group and BPV + LE + TAT-Pep5 group (*P* < 0.05). Compared with the BPV group, both the LE group and the BPV + LE + K252a group showed increased p75^NTR^ protein expression (*P* < 0.05), while the BPV + LE group and the BPV + LE + TAT-Pep5 group showed increased p75^NTR^ protein expression (*P* > 0.05).

The cleaved caspase-3 protein changes in the six groups of hippocampal neurons are shown in Fig. 5, E1–E2. Cleaved caspase-3 protein expression was increased in the BPV group compared with the control group (*P* < 0.05), while compared with the BPV group, cleaved caspase-3 protein expression in the BPV + LE group was decreased (*P* < 0.05). Cleaved caspase-3 protein expression was decreased in the BPV + LE + TAT-Pep5 group compared with the BPV + LE group (*P* < 0.05). Cleaved caspase-3 protein expression was decreased in the BPV + LE + TAT-Pep5 group compared with the BPV + LE + K252a group (*P* < 0.05) (Fig. 5).

## Discussion

These experiments used hippocampal neurons as the research object to study LE treatment’s impact on the toxic effects of BPV on the CNS. The in vitro experiment eliminated body metabolism and adjusted for factors, such as interference, and validated that LE treatment was effective in reducing hippocampal neuron cell toxicity.

Studies have shown that the neurotoxicity of all local anaesthetics is concentration and time-dependent^25,26^. We tested the influence of BPV on hippocampal neurons and measured its IC50, which was 980.4 μM. In our experiments, 1000 μM or 1 mM BPV inhibited 50% of hippocampal neuron activity after 24 h of treatment. Therefore, this concentration and treatment time were used in the following experiments. However, our study sought to answer the following question: can LE prevent BPV-induced reductions in hippocampal neuronal activity? We chose three LE concentrations of 0.5%, 1%, and 2% and observed their effects on hippocampal neuron cell vitality. We found that the three concentrations of LE had no obvious inhibitory effect on hippocampal neuronal activity. We also found that the 1% and 2% concentrations caused a slight increase in hippocampal neuronal activity. Combined with previous studies on treating BPV toxicity with LE^14,15^, the effects of 1 mM BPV and 1% LE on BPV-induced neurotoxicity in hippocampal neurons were studied after 24 h of treatment with LE. In this study, the concentrations of the TrkB inhibitor K252a and the p75^NTR^ inhibitor TAT-Pep5 were referenced from the literature^27,28^.

We observed under the optical microscope that BPV inhibited the growth of hippocampal neurons. Compared with the BPV group, hippocampal neuron projections in the BPV + LE group were increased, with the surrounding hippocampal neurons interwoven into a network, and the connection between hippocampal neurons increased. This also confirmed that local anaesthetic can inhibit the growth of a neuron’s synaptic axon^29^, and LE treatment can reduce neurotoxic effects and promote both the growth of hippocampal neurons and the connections between them.

Information transmission and functional connections between hippocampal neurons are mainly mediated by synaptic activity. BDNF and its precursor proBDNF are secreted by neuron cell bodies to stimulate TrkB and p75^NTR^ receptors located on axons and dendrites, as well as other receptors involved in neuron development and synaptic plasticity (SP)^30,31^. However, studies on the role of the BDNF-TrkB pathway have different results. BDNF promotes neuronal survival by activating TrkB in different brain regions and provides a neuroprotective effect^32,33^. The BDNF-TrkB-dependent ERK pathway is involved in the process of combating glutamate excitatory neurotoxicity and plays a neuroprotective role^28,34^. It has also been reported that BDNF can stimulate glutamate release by activating TrkB and downstream pathways^35^. The BDNF-TrkB pathway is involved in the development of epilepsy, depression, Alzheimer’s disease, and other diseases^36,37^. Blocking this pathway can be used as a therapeutic target for such diseases. In nerve endings, BDNF promotes glutamate release and inhibits the release of γ-aminobutyric acid (GABA) through a variety of mechanisms^35,38^. Glutamate-induced excitatory toxicity of hippocampal neurons is associated with the downregulation of full-length TrkB receptors and upregulation of truncated TrkB receptors^32^. Many studies have reported that the proBDNF-p75^NTR^ pathway and the BDNF-TrkB pathway have opposite biological roles in promoting neuron survival and SP^37,39–42^.

In this experiment, the protein expression and mRNA levels of TrkB and p75^NTR^ were decreased when bupivacaine induced hippocampal neuronal toxicity, while the expression of BDNF was increased and proBDNF showed no significant change, which was inconsistent with the previous experimental results of neuronal toxicity. This result may be related to the experimental parameters and drugs. Previous studies have confirmed BDNF-TrkB and related pathways downstream of its biological function, most of which adopt methods such as inducing excitatory neurotoxicity by glutamate, creating epileptic convulsions or depression models, or increasing exogenous BDNF. Research reports show that epileptic activity increases the expression of BDNF^43,44^, and by activating TrkB, BDNF can play a role in neural protection. Therefore, we consider that hippocampus induced neurotoxicity increased BDNF protein expression because hippocampal neurons were noxiously stimulated by bupivacaine and reflexively increased the expression of BDNF, resulting in a self-protective effect. LE increased TrkB protein expression and mRNA levels, activated BDNF, promoted hippocampal neurite growth, and mediated neuroprotective effects. BPV itself exerts a biological effect by preventing the intracellular flow of Na^+^ required by neurons to generate an action potential and inhibits the transmission of intersynaptic transmitters^29,45^, as well as the growth of synaptic axons. This may be the reason for the downregulated expression of receptor p75^NTR^ protein and mRNA levels on the dendrites and axons of hippocampal neurons. The expression levels of p75^NTR^ protein and mRNA in the BPV + LE group were slightly increased compared with those in the BPV group, which may be related to the protective effect of LE in hippocampal neuronal processes.

BPV can induce apoptosis in nerve cells^46,47^. Apoptosis plays an essential role in the removal of unwanted or abnormal cells by multicellular organisms, and is a basic measure to maintain the dynamic balance of cell numbers in vivo. Disorders of apoptosis processes may be directly or indirectly related to the occurrence of many diseases. Caspases (cysteinyl aspartate specific proteinases) are a group of proteases involved in cell growth, differentiation, and apoptosis regulation, and are closely related to eukaryotic cell apoptosis. Caspase is responsible for selectively cleaving certain proteins to cause apoptosis, and Caspase-3 is the ultimate executor of apoptosis^48^. Cleaved caspase-3, an activated form of caspase-3, is often used as a marker of apoptotic cells^49^.

For cleaved caspase-3 half quantitative analysis of protein content, the results showed that BPV increased the original generation of cleaved caspase-3 protein content by hippocampal neurons, and LE and BPV decreased the cleaved caspase-3 protein content of hippocampal neurons, showing that BPV induced hippocampal neuron apoptosis, whereas LE could reduce the apoptosis of hippocampal neurons induced by BPV, which is associated with the cell protective effects of BDNF-TrkB. However, after K252a inhibited TrkB activity, hippocampal neuron apoptosis was not statistically significant compared with that in the BPV + LE group, which may be related to decreased TrkB expression. It has been reported in the literature that K252a was not sensitive to BDNF's inhibition of the downstream excitatory cell death pathway^32^, and thus, the effect of LE was not significantly inhibited. After tat-pep 5 inhibited the activation of the proBDNF-p75^NTR^ pathway, the effect of LE was significantly enhanced, and hippocampal neuron apoptosis was significantly reduced. It was speculated that LE could reduce the hippocampal neuron apoptosis caused by BPV by inhibiting the activity of the proBDNF-p75^NTR^ pathway.

In conclusion, this study confirmed that LE may reduce hippocampal neuron apoptosis by regulating the BDNF-Trkb and probBDNF-p75^NTR^ pathways to promote the survival of hippocampal neurons and reduce the toxicity of rat hippocampal neurons induced by BPV, providing a basis for the treatment of BPV CNS toxicity by LE in the future.

### Supplementary Information

Supplementary Information.

## Data Availability

The datasets used and analyzed during the current study are available from the corresponding author on reasonable request.

## Abbreviations

BCA: Bicinchoninic acid
BDNF: Brain-derived neurotrophic factor
BPV: Bupivacaine
BSA: Bovine serum albumin
CNS: Central nervous system
EDTA: Ethylene Diamine Tetraacetic Acid
GABA: γ-Aminobutyric acid
IC50: Half maximal inhibitory concentration
LE: Lipid emulsion
p75^NTR^: P75 neurotrophic factor receptor
PVDF: Polyvinylidene fluoride
SDS-PAGE: Sodium dodecyl sulfate polyacrylamide gel electrophoresis
TrkB: Tyrosine kinase receptor B
