# Supplementary figures and images for: BDNF-TrkB/proBDNF-p75NTR pathway regulation by lipid emulsion rescues bupivacaine-induced central neurotoxicity in rats
Source: Sci Rep. 2023 Oct 26;13:18364. doi: 10.1038/s41598-023-45572-8 (PMC10603093; doi:10.1038/s41598-023-45572-8)

BDNF  
(1)

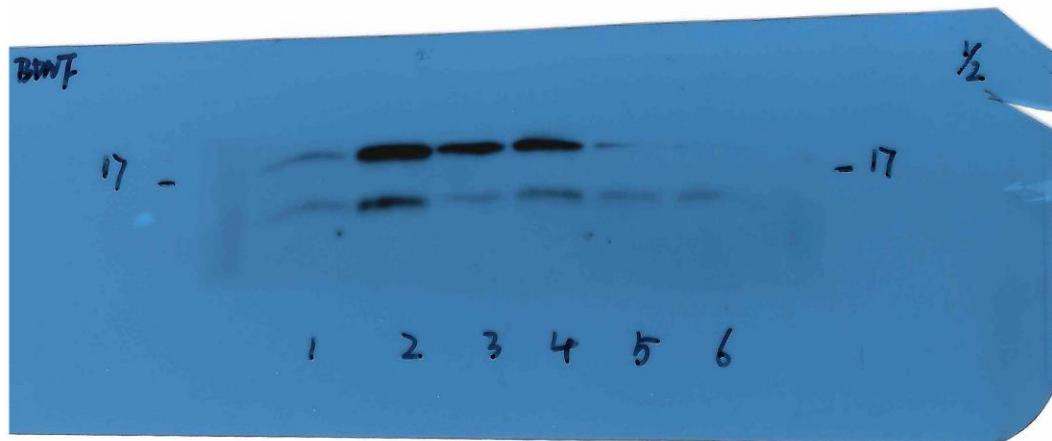

(2)

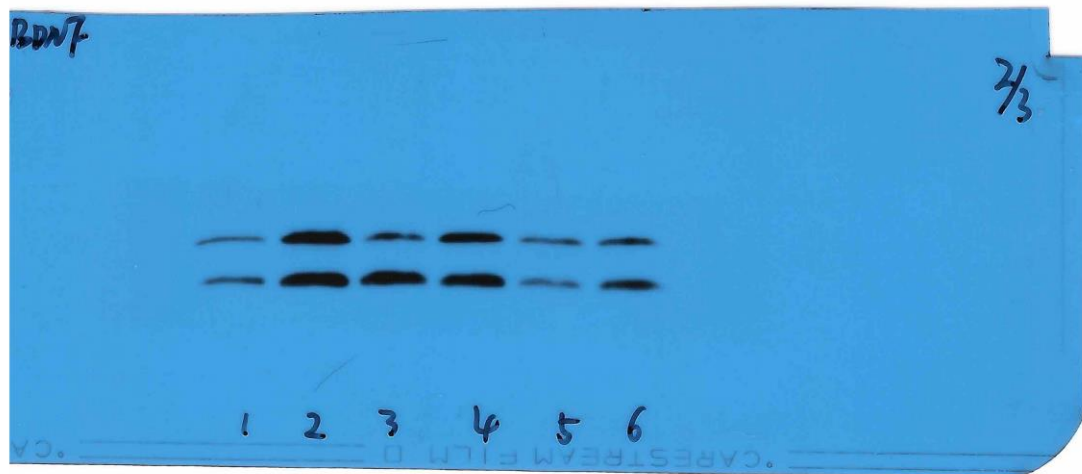

(3)

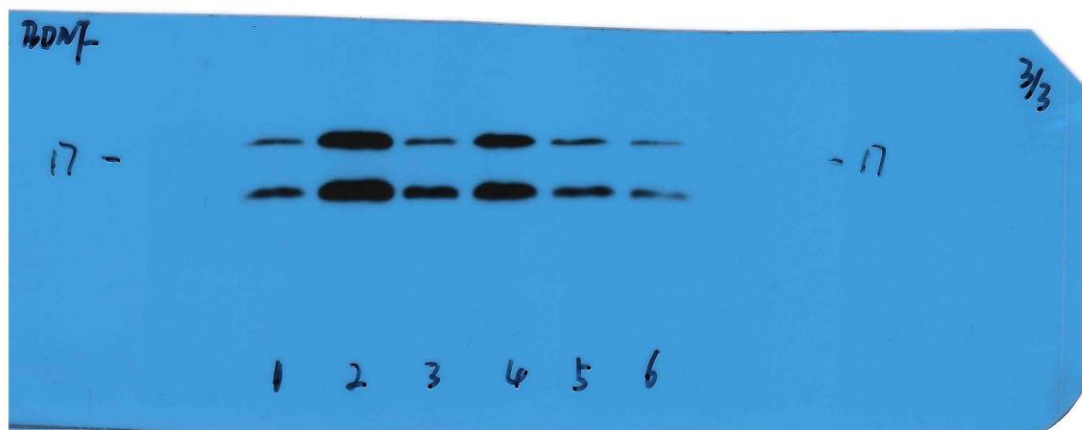

$\beta$ -actin  
(1)

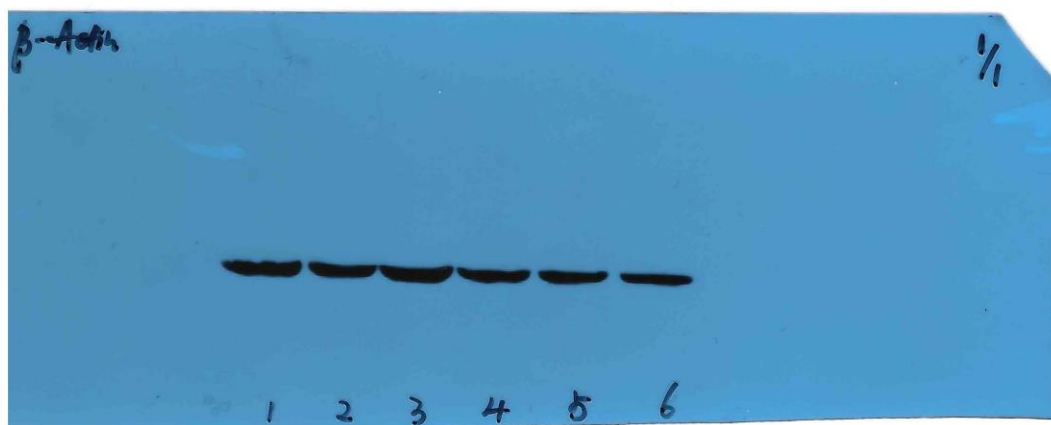

(2)

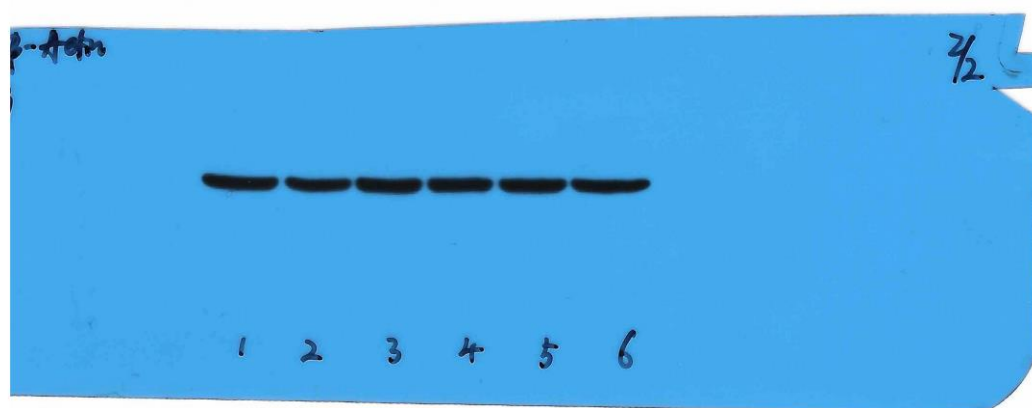

(3)

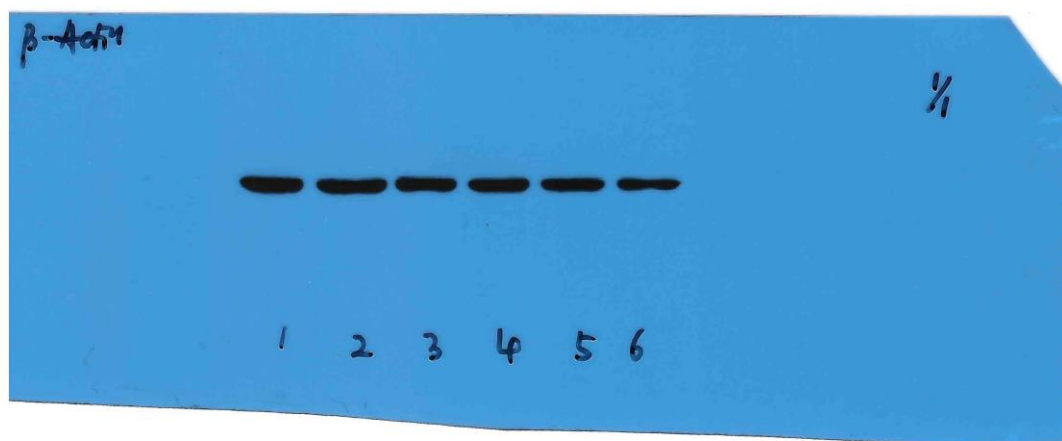

proBDNF

(1)

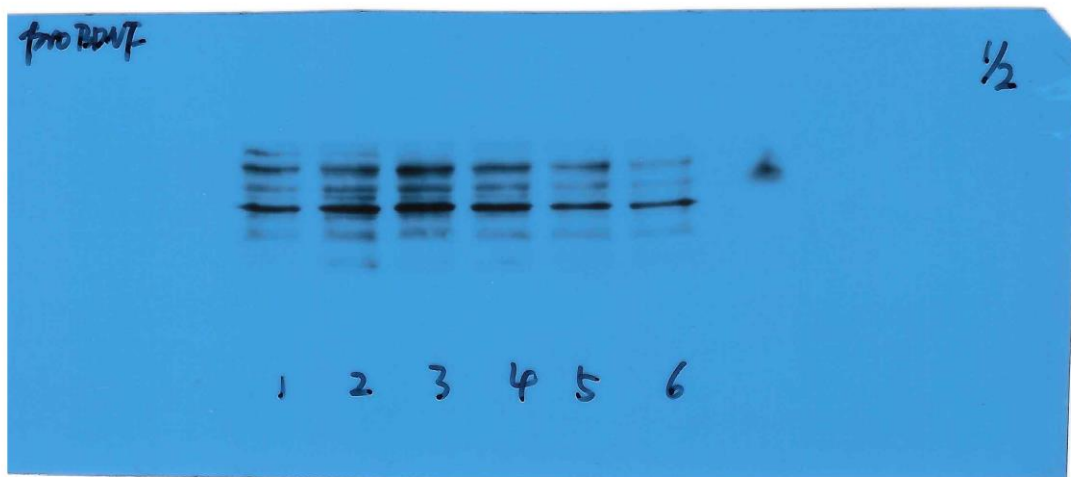

(2)

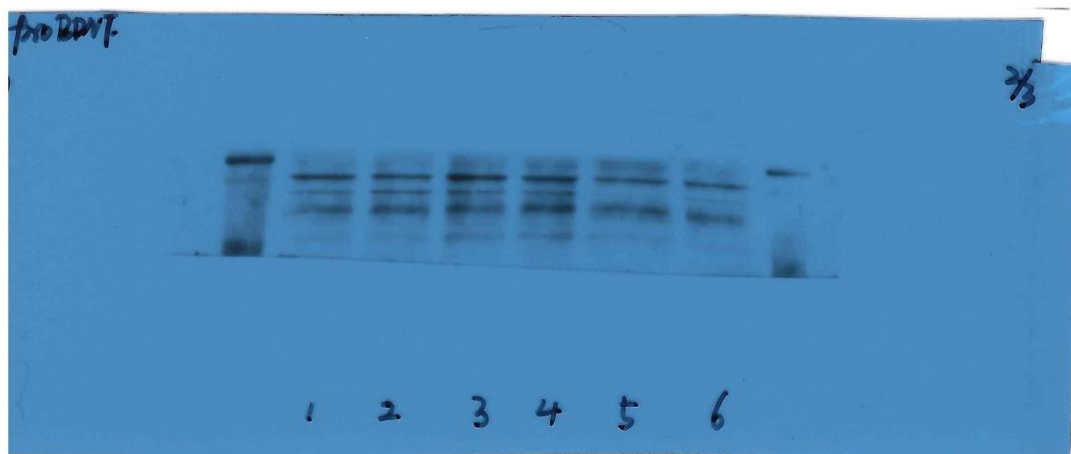

(3)

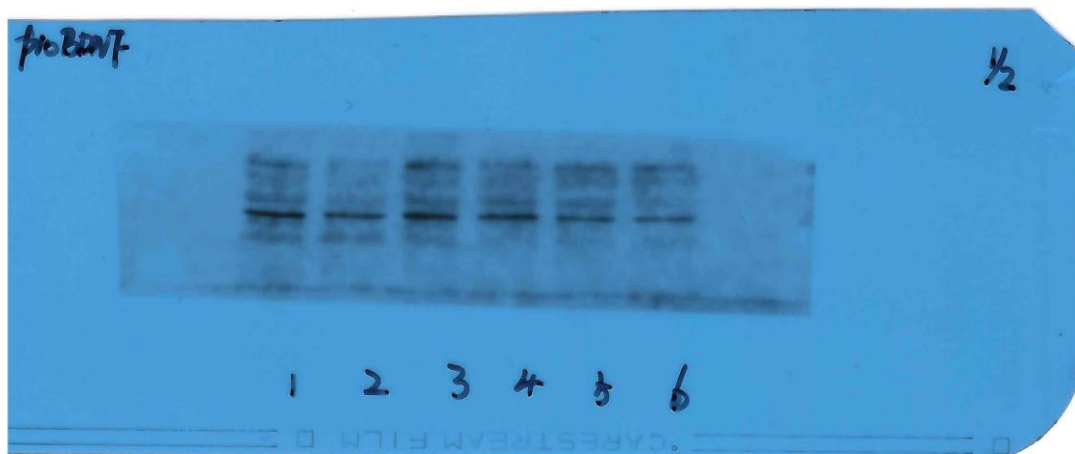

β-actin

(1)

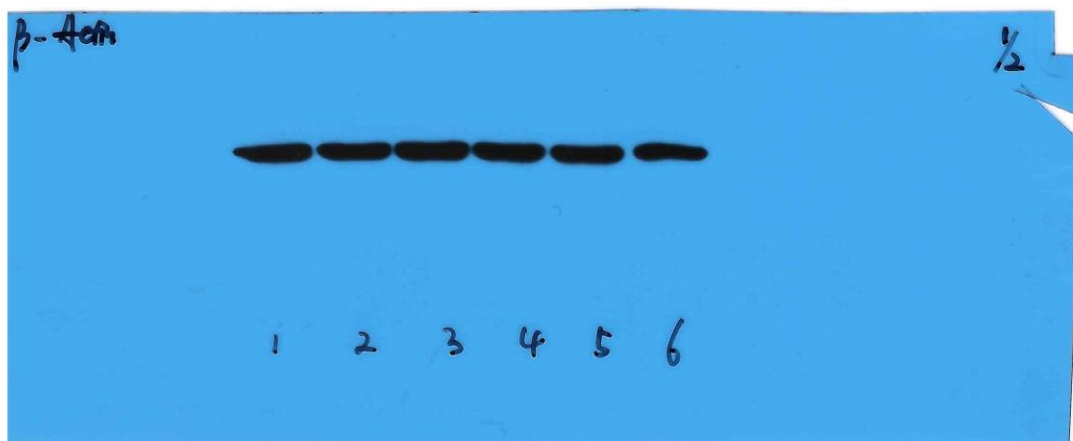

(2)

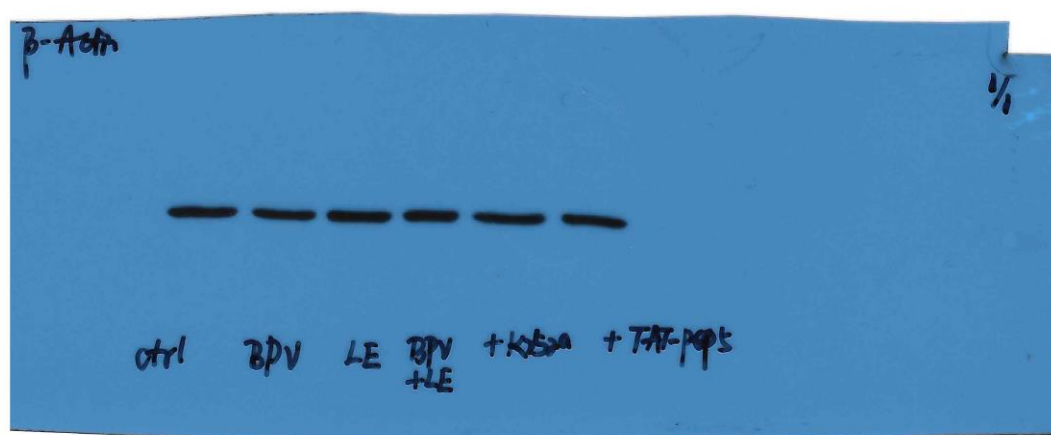

(3)

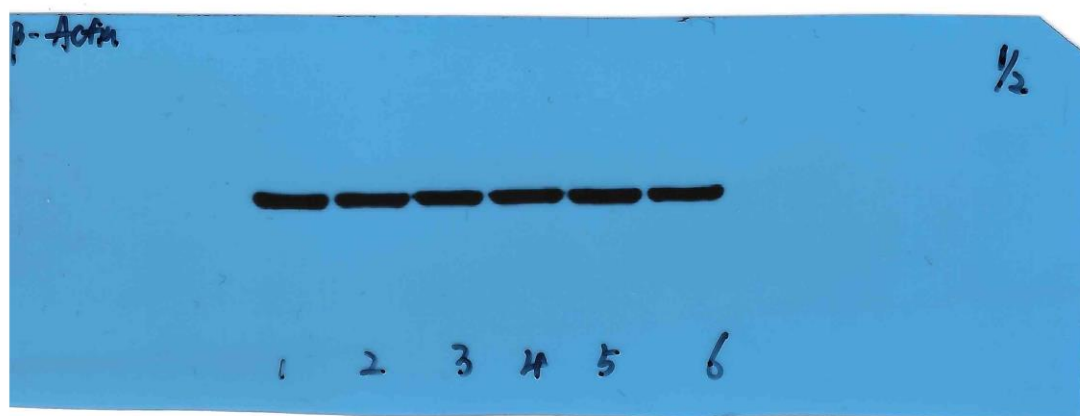

TrkB

(1)

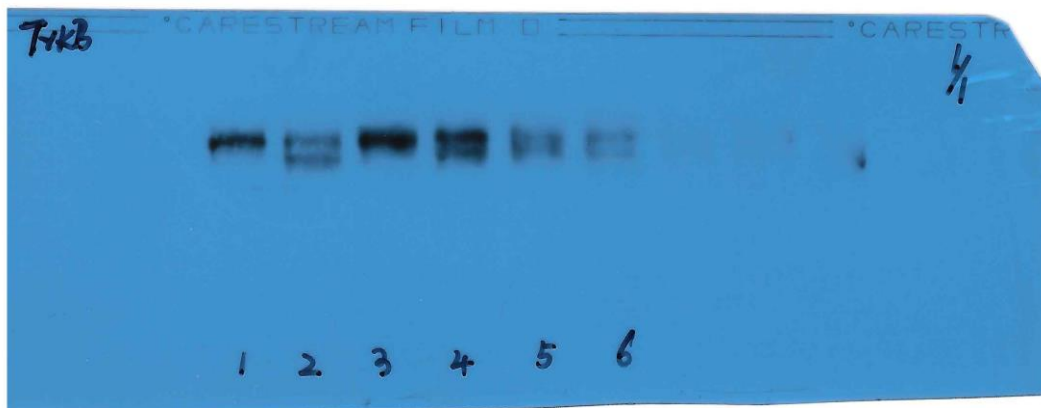

(2)

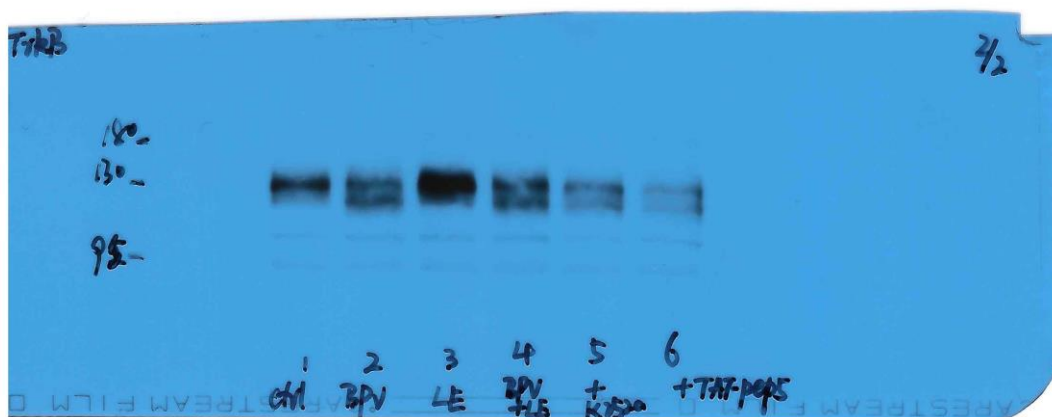

(3)

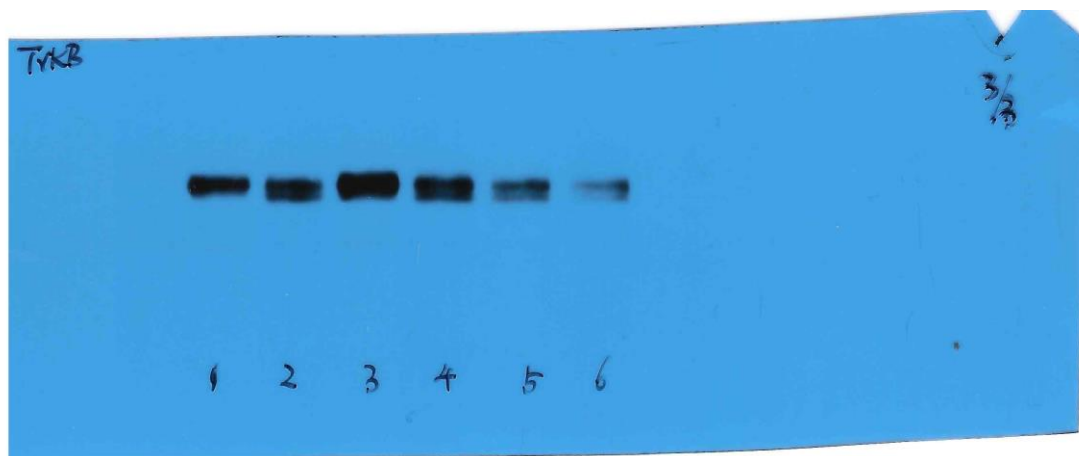

$\beta$ -actin

(1)

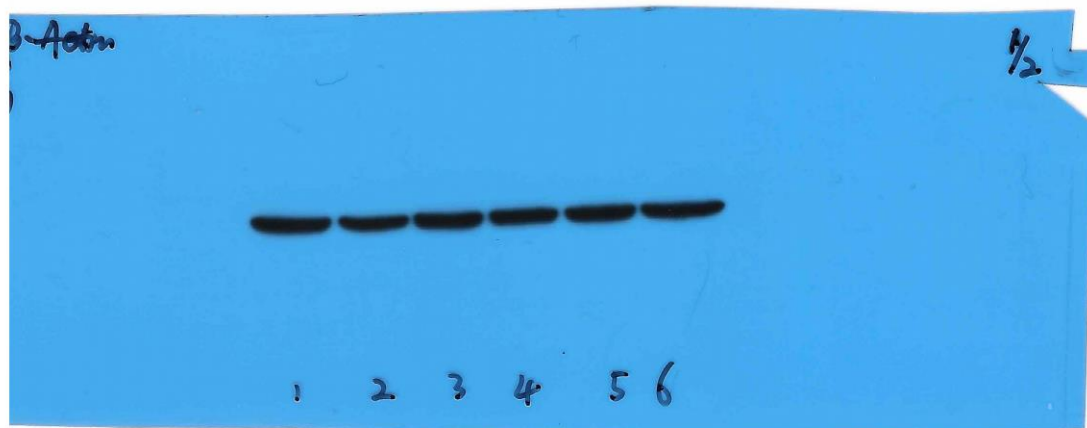

(2)

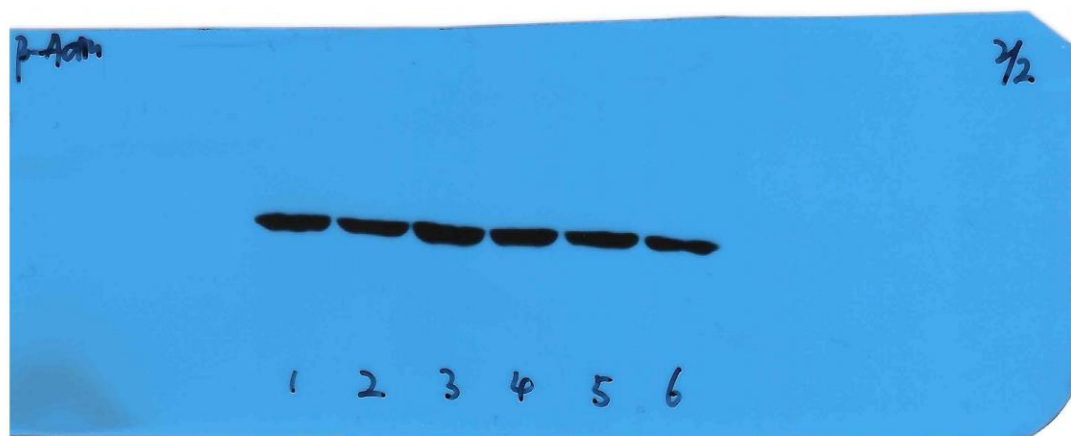

(3)

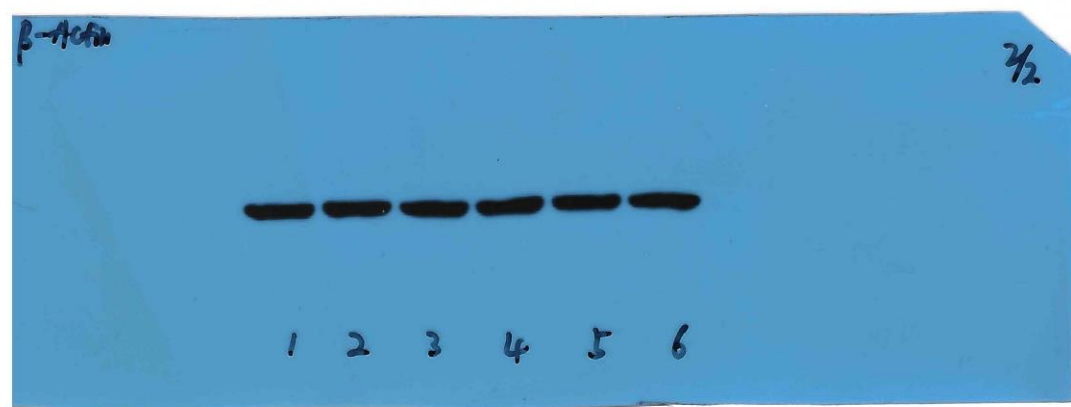

p75<sup>NTR</sup>  
(1)

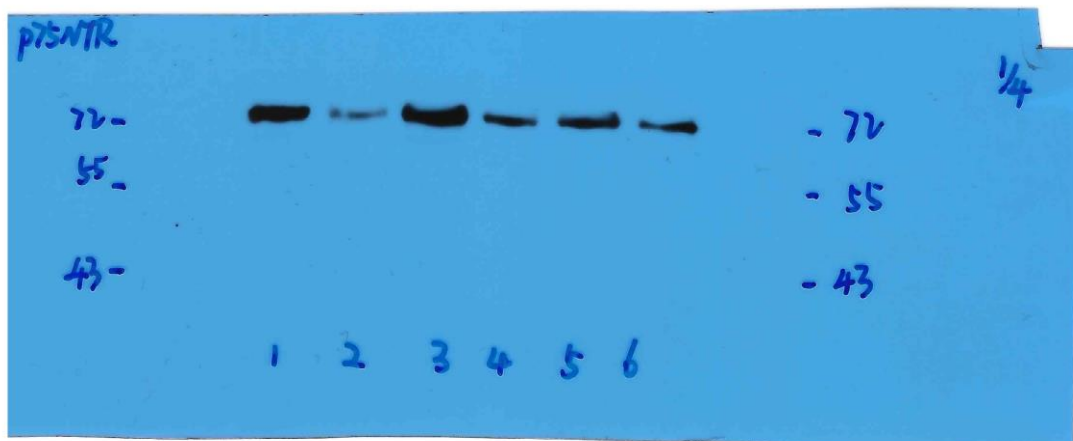

(2)

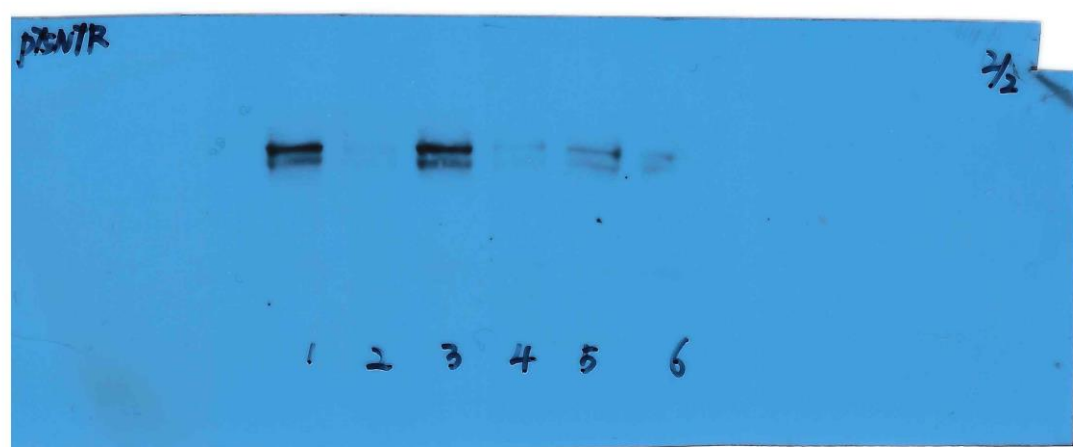

(3)

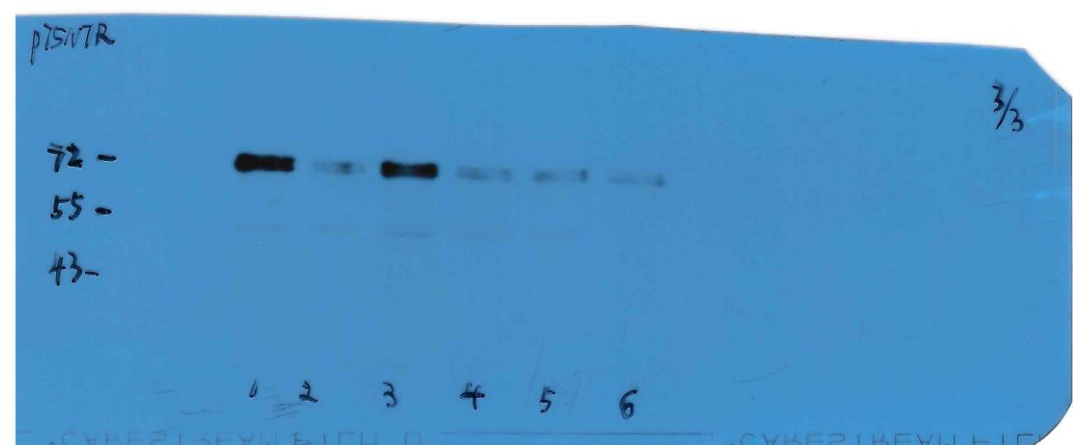

β-actin

(1)

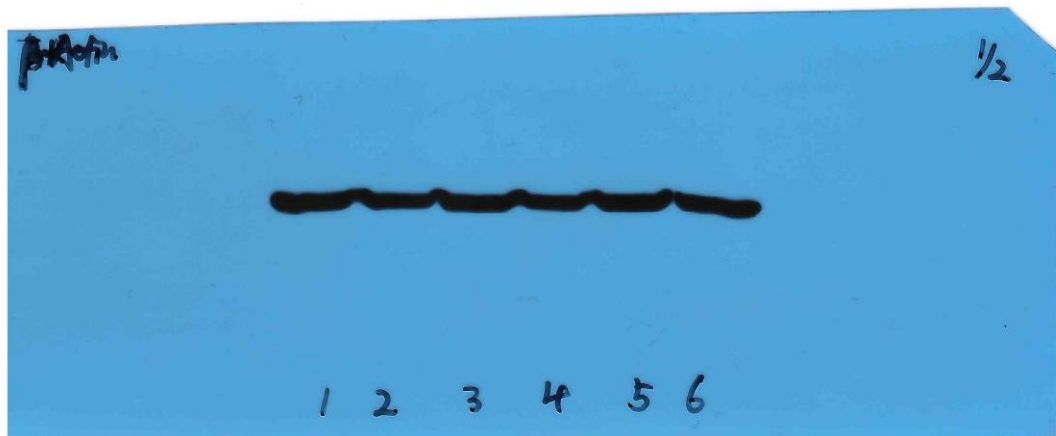

(2)

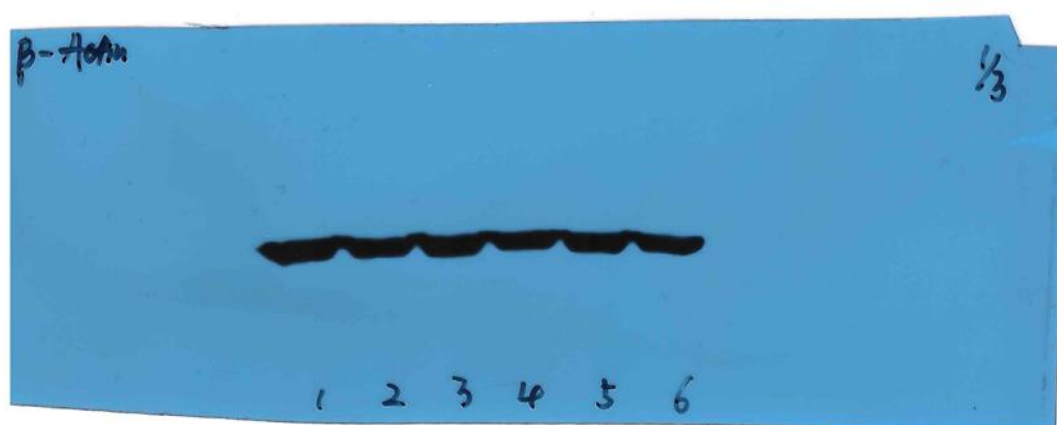

(3)

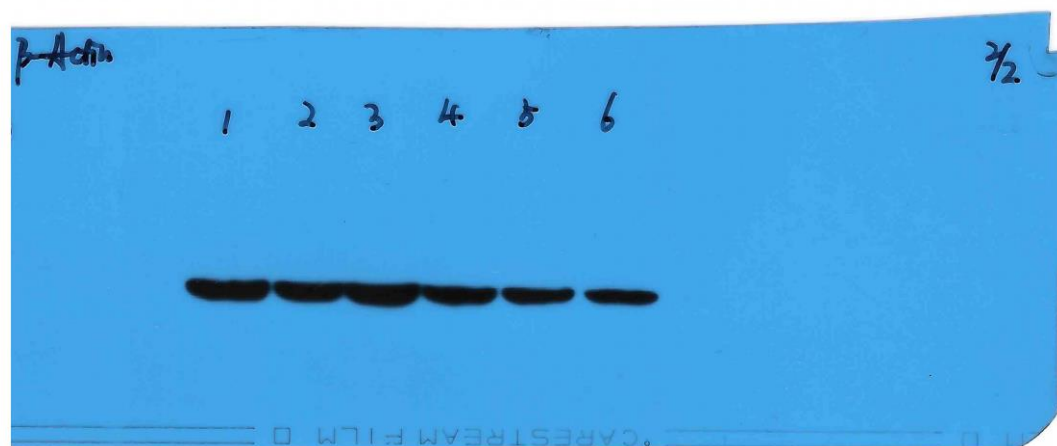

Cleaved Caspase-3

(1)

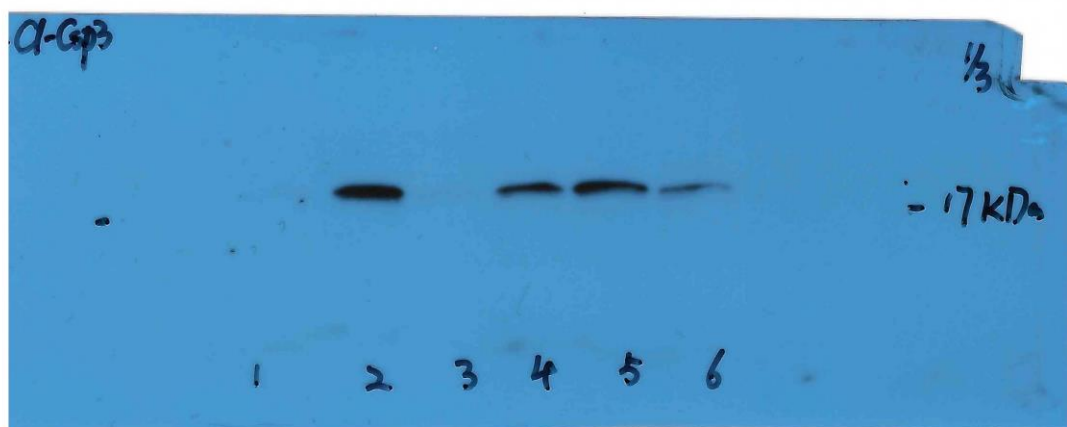

(2)

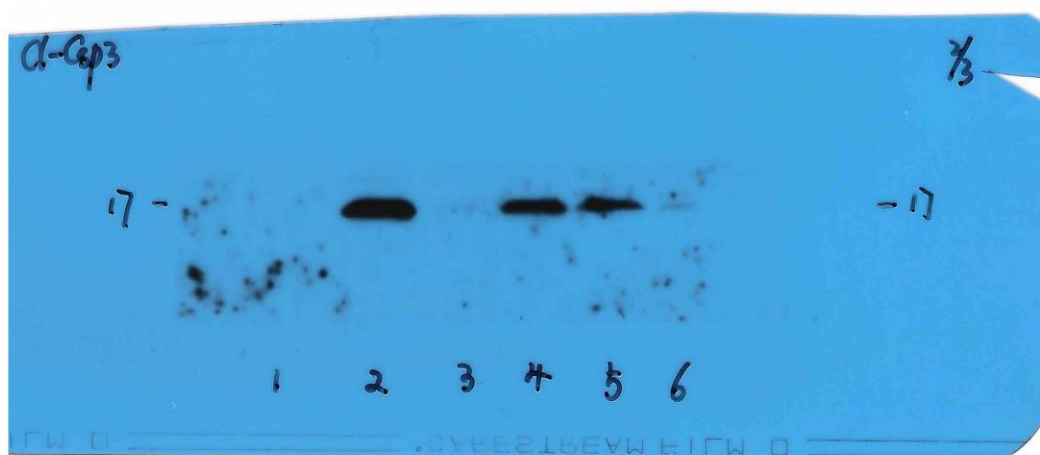

(3)

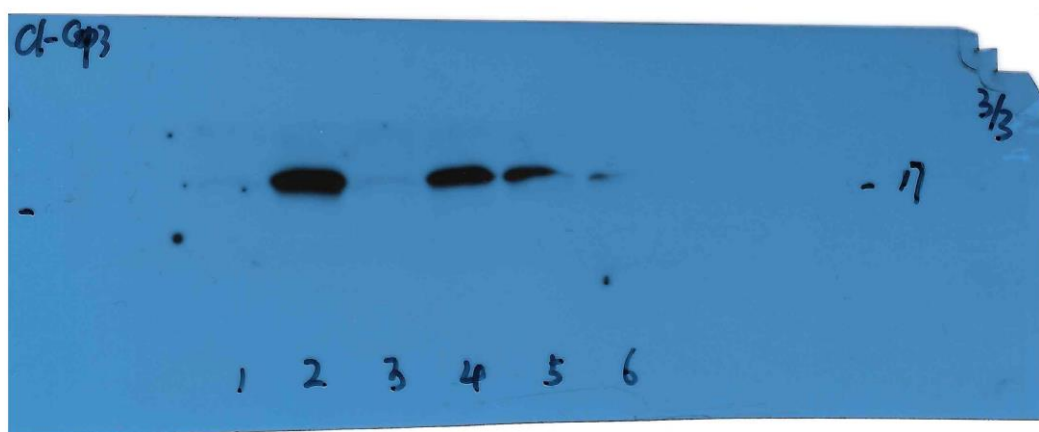

$\beta$ -actin

(1)

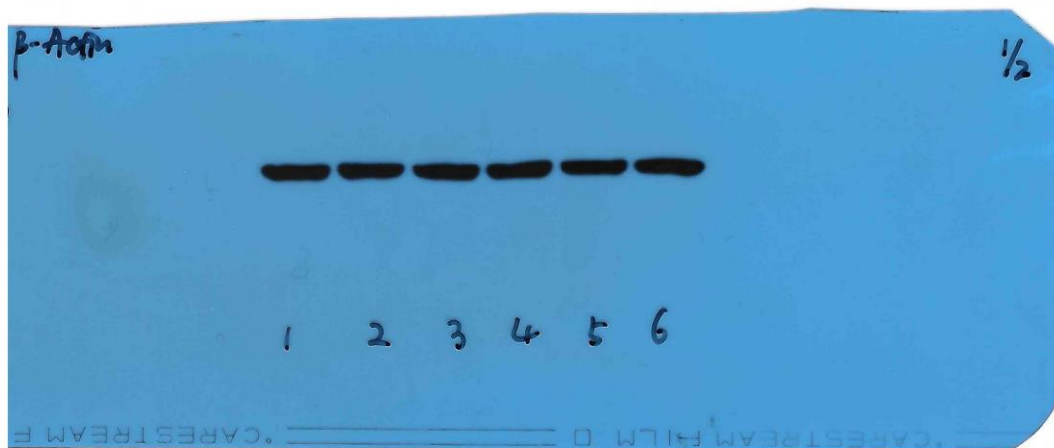

(2)

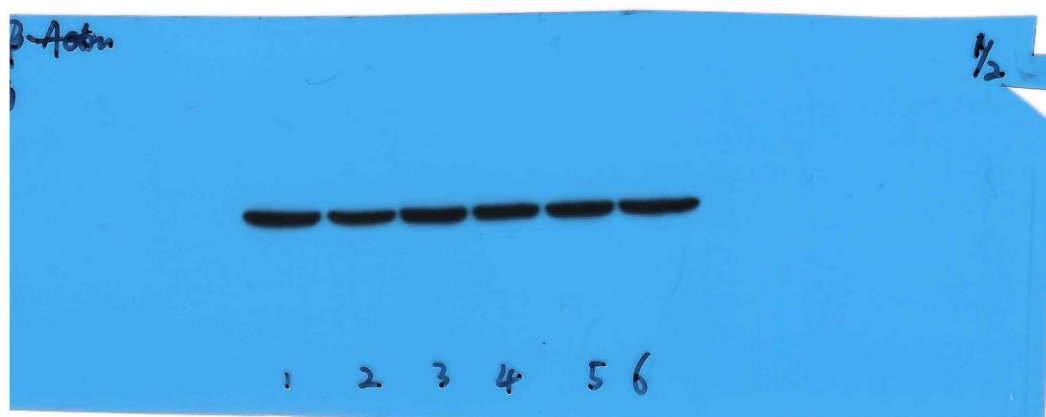

(3)

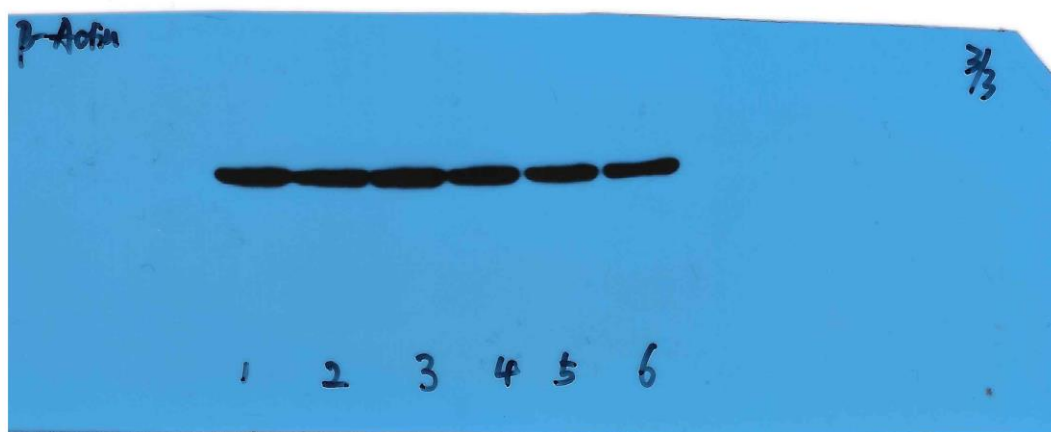

Supplement: Supplementary file 1 — Supplementary Information. [file 41598_2023_45572_MOESM1_ESM.pdf]
